# Do the psychological effects of vagus nerve stimulation partially mediate vagal pain modulation?

**DOI:** 10.1016/j.ynpai.2017.03.002

**Published:** 2017-03-31

**Authors:** Eleni Frangos, Emily A. Richards, M. Catherine Bushnell

**Affiliations:** National Center for Complementary and Integrative Health, National Institutes of Health, Bethesda, MD 20892, United States

**Keywords:** ABVN, auricular branch of the vagus nerve, ACC, anterior cingulate cortex, DMN, default mode network, FDA, Food and Drug Administration, fMRI, functional magnetic resonance imaging, GABA, gamma-aminobutyric acid, IL, interleukin, iVNS, invasive vagus nerve stimulation, MDD, major depressive disorder, NTS, nucleus tractus solitarius, PAG, periaqueductal gray, STN, spinal trigeminal nucleus, TNFα, tumor necrosis factor alpha, VNS, vagus nerve stimulation, tVNS, transcutaneous vagus nerve stimulation, Vagus nerve, Pain, Affect, tVNS

## Abstract

There is preclinical and clinical evidence that vagus nerve stimulation modulates both pain and mood state. Mechanistic studies show brainstem circuitry involved in pain modulation by vagus nerve stimulation, but little is known about possible indirect descending effects of altered mood state on pain perception. This possibility is important, since previous studies have shown that mood state affects pain, particularly the affective dimension (pain unpleasantness). To date, human studies investigating the effects of vagus nerve stimulation on pain perception have not reliably measured psychological factors to determine their role in altered pain perception elicited by vagus nerve stimulation. Thus, it remains unclear how much of a role psychological factors play in vagal pain modulation. Here, we present a rationale for including psychological measures in future vagus nerve stimulation studies on pain.

## Introduction

Modulation of pain perception via vagus nerve stimulation (VNS) has been examined in humans and animals for several years using an invasive approach (iVNS; Randich and Gebhart, 1992, Yuan and Silberstein, 2016), and more recently, with non-invasive transcutaneous approaches (tVNS; Busch et al., 2013, Nesbitt et al., 2015). While a number of mechanistic studies in animals have uncovered portions of the mechanism of action underlying vagal-induced pain modulation (Randich and Gebhart, 1992, Nishikawa et al., 1999), the entirety of the mechanism in humans remains elusive. Optimal stimulation durations and parameters, and determining which pain modalities are most responsive have yet to be discerned.

Pain perception and modulation, outside of vagus nerve stimulation, is complex. The sensory (nociceptive) and affective components of pain can vary between and within individuals, and the latter psychological aspect of pain weighs heavily on the manner in which pain is perceived (Bushnell et al., 2013). Moreover, cognitive processes (e.g., attention) compared to emotions and mood states differentially modulate the intensity and unpleasantness of pain (Villemure et al., 2003, Loggia et al., 2008, Villemure and Bushnell, 2009). Despite evidence that VNS improves affect, as it is used therapeutically against depression, studies investigating vagal modulation of pain primarily focus on obtaining measures of pain threshold or the sensory (nociceptive) component of pain as measured by pain intensity ratings. Key psychological factors that affect pain such as mood state and attention are not reliably being measured. Thus, it remains unclear as to whether the effects of VNS on pain are, in part, due to modulation of not only the sensory component of pain perception, but also the psychological component.

Here, we discuss the effects of 1) *VNS on pain perception*, 2) *psychological factors on pain perception*, and 3*) VNS on psychological factors.* The known mechanisms of vagal pain modulation are summarized (4), and a proposed model of vagal pain modulation is presented (5) as an impetus for future studies to include psychological measures when examining the effects of VNS on pain perception.

## The effects of VNS on pain perception

### iVNS modulation of pain perception

Invasive vagus nerve stimulation (iVNS) is a current treatment option for patients with refractory epilepsy and depression. This method of treatment requires an implanted device that provides direct electrical stimulation to the left cervical vagus nerve. The pain relieving effects of vagus nerve stimulation in humans were first observed in patients receiving iVNS for either epilepsy or depression. Incidentally, patients suffering from concomitant migraine or cluster headache reported decreases in frequency and severity of attacks or complete relief after the iVNS implant (Kirchner et al., 2000, Sadler et al., 2002, Hord et al., 2003, Lenaerts et al., 2008), suggesting that iVNS could alter pain in not only animals, as had already been demonstrated, but also in humans. A summary of case reports on six patients who received an iVNS implant to specifically treat migraine and cluster headache concluded that iVNS might be an effective therapy for these conditions as improvement levels in four patients ranged from good to excellent (Mauskop, 2005). Similar beneficial effects were observed in patients receiving iVNS for the treatment of chronic daily headache and depression (Cecchini et al., 2009). A more recent proof-of-concept trial found that iVNS may be effective in treating fibromyalgia, as seven of 14 patients progressively attained a *minimal clinically important difference* after 11 months of iVNS treatment. Furthermore, two of the seven patients no longer fulfilled the diagnostic criteria for fibromyalgia. The patients also experienced a decrease in pain ratings to noxious heat stimuli before vs. after iVNS implantation and over the course of 11 months (Lange et al., 2011).

In epilepsy patients, Kirchner et al. (2000) reported a decrease in temporal summation of pain (wind-up) and tonic pressure pain independent of the iVNS on/off cycle after 8–14 weeks of iVNS compared to before implantation. One patient in this study suffering from chronic tension-type headache for more than 10 years experienced an 80% reduction of headache after the surgery. In a later study, Kirchner et al. (2006) successfully replicated their findings for tonic pressure pain and also observed a limited but significant inhibitory effect on neurogenic inflammation (axon reflex flare) produced by tonic mechanical pressure (pinching of the fingerfolds) after iVNS implantation. Conversely, Ness et al. (2000) reported significant decreases in thermal pain thresholds in epilepsy patients in response to individual optimal and suboptimal iVNS intensities compared to sham. The increased sensitivity to pain in response to the low intensity iVNS corroborated earlier reports in rats demonstrating that low intensity iVNS induces pro-nociceptive effects in response to heat, whereas high intensity iVNS induced analgesic effects (Ren et al., 1988). The differential parameter effects are discussed further in Section “Mechanisms of vagal modulation of pain”. That said, in a subsequent communication, Ness et al. (2001) provided thermal wind-up results similar to those observed in the Kirchner et al. (2000) study, thereby supporting a central pain inhibitory mechanism.

In one case, a patient receiving iVNS for chronic depression experienced complete relief from both depression and chronic back pain after 35 months of iVNS at an optimal setting, but reported an increase in pain intensity ratings in response to acute noxious thermal stimuli during the *on* phase of iVNS compared to the *off* phase (Borckardt et al., 2006)*.* Similarly, depressed patients receiving iVNS with different combinations of parameter settings experienced reduced tolerance to painful heat (Borckardt et al., 2005). However, it is difficult to draw conclusions from this study given the few number of participants, the various device settings that potentially render it underpowered, the prevalence of comorbid chronic pain, and the short duration of iVNS stimulation at low intensities. Nevertheless, the measurable changes detected lend support towards vagal modulation of pain despite the undesirable direction. Interestingly, contrary to the experimental findings, two of the participants anecdotally reported relief of their preexisting pain conditions after iVNS implantation. In one of the cases, the patient with chronic low back pain reported he was no longer “bothered” by the pain after implantation. It remains unclear if the intensity of his back pain changed, but evidently the negative affective component of the pain was reduced.

Table 1 summarizes the studies described above. Eight of the 11 iVNS studies discussed here reported significant reductions in pain perception, which demonstrates a potential beneficial impact of iVNS on evoked pain and chronic pain conditions such as headache and low back pain. The studies have limitations such as small sample sizes and the existing pathology can be a confounding factor. Indeed, an inverse correlation between the severity of depression and pain tolerance in iVNS patients has been reported (Borckardt et al., 2005). Some of these limitations, such as the inclusion of proper controls and testing on healthy participants, can now be addressed with non-invasive VNS devices.

**Table 1:** Summary of invasive vagus nerve stimulation (iVNS) studies.

Author	Sample	Parameters	Main outcomes	
			Pain	Affect
Borckardt et al. (2005)	Depression n = 8	Combination of: 20 Hz/30 Hz; 130 μs/250 μs/500 μs pw at 0%, 50%, 100% of baseline intensity (0–2.75 mA)	↓ heat tolerance	Not reported
Borckardt et al. (2006)	Depression/low back pain n = 1	10 Hz or 20 Hz, 250 μs or 500 μs pw 0–0.75 mA 30 s on, 3–20 min off	↓ low back pain ↑ experimental pain ratings ns pain thresholds	↓ depression
Cecchini et al. (2009)	Migraine n = 4	30 Hz, 500 ms pw1-2.25 mA 30 s on, 5 min off	↓ migraine	↓ depression
Hord et al. (2003)	Migraine n = 4	30 Hz, 500 ms pw ∼1.0 mA 30 s on, 5 min off	↓ migraine frequency/intensity	Not reported
Kirchner et al. (2000)	Epilepsy n = 10 Healthy n = 12	30 Hz, 500 μs pw initial: 0.7 ± 0.2 mA final: 1.4 ± 0.3 mA	↓ wind-up, pressure pain in patients, comorbid migraine ns thermal, mechanical pain	Not reported
Kirchner et al. (2006)	Epilepsy n = 9 Healthy n = 9	For majority: 20 Hz, 500 μs pw 30 s on, 30 s off	↓ tonic pressure pain in patients ↓ axon reflex flux	Not reported
Lange et al. (2011)	Fibromyalgia n = 14	20 Hz, 250 μs pw, 1–2 mA 30 s on, 5 min off	↓ fibromyalgia symptoms ↓ heat pain sensitivity ↓ pain intensity	Not reported
Lenaerts et al. (2008)	Migraine n = 10	Not reported	↓ migraine frequency	↓ (ns) mood/anxiety
Mauskop (2005)	Migraine n = 6	250 ms pw, 1.25–2.75 mA 7–60 s on, 0.2–5 min off,	↓ migraine/frequency	↓ prodromal depression
Ness et al. (2000)	Epilepsy n = 8	30 Hz, 0.5 ms pw 1.0–2.75 mA 30 s on, 3–5 min off	↓ heat pain thresholds	Not reported
Sadler et al. (2002)	Epilepsy/Migraine n = 1	Final: 20 Hz, 250 μs pw 0.25 mA 7 s on, 12 s off	↓ migraine frequency	Not reported

↓, decrease.

↑, increase.

ns, non-significant.

pw, pulse width.

### Non-invasive VNS modulation of pain perception

**Fig. 1:**
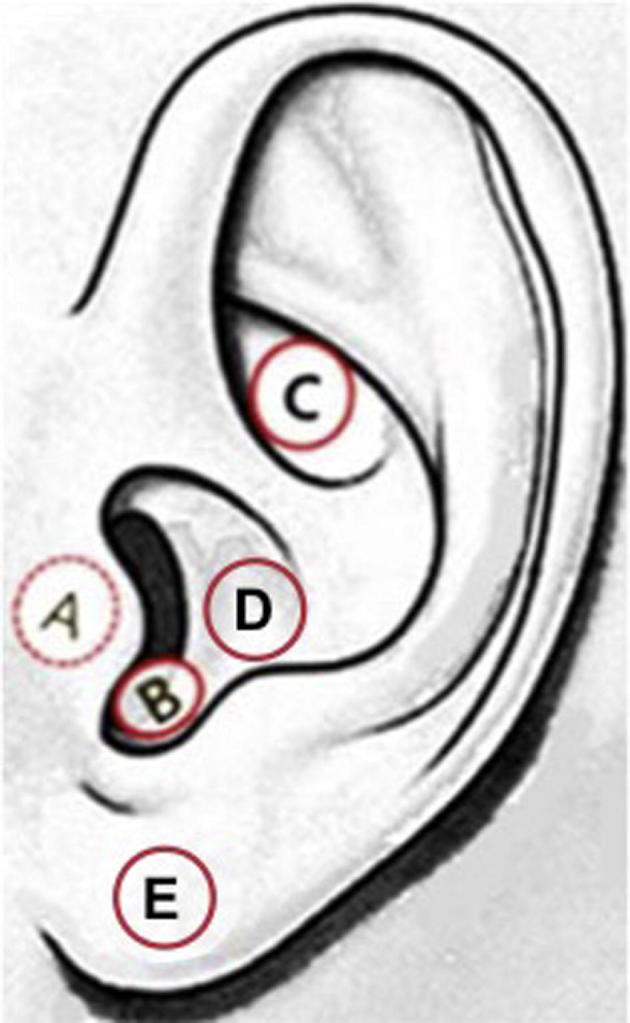
The left external ear indicating the regions where tVNS has been applied (A-D) and where control stimulation has been applied (E). A: inner side of the tragus, B: anterior, posterior and/or inferior walls of the ear canal, C: cymba conchae, D: cavum conchae, E: earlobe. (This figure is a modification of Fig.1c. found in Yakunina et al., 2016).

The recent development of transcutaneous (non-invasive) vagus nerve stimulation (tVNS) has made it feasible to test vagal stimulation on not only patients, but healthy participants as well. This non-invasive approach requires mild electrical stimulation of regions of the external surface of the ear that are innervated by the auricular branch of the vagus nerve (ABVN), namely, the posterior and inferior walls of the ear canal, the inner side of the tragus, the cavity of the concha, or the cymba conchae (Fay, 1927, Peuker and Filler, 2002). The cymba conchae region of the ear is the only region of the ear exclusively innervated by the ABVN (Peuker and Filler, 2002), and has been identified as the optimal location for auricular tVNS (Yakunina et al., 2016). Tract-tracing studies in animals and fMRI studies in humans provide evidence that the AVBN projects to the nucleus of the solitary tract (NTS), the location of the first central relay of cervical vagal afferents in the medulla region of the brainstem (Nomura and Mizuno, 1984, Berthoud and Neuhuber, 2000, Frangos et al., 2015, Yakunina et al., 2016). Fig. 1 depicts the various regions of the ear that have been stimulated in order to gain access to the ABVN; in some cases, the earlobe has been used as an active control.

Although comparative studies have yet to be conducted, auricular tVNS is exhibiting similar beneficial effects on drug-resistant epilepsy and treatment-resistant depression as observed with iVNS (Stefan et al., 2012, Hein et al., 2013, Aihua et al., 2014, Bauer et al., 2016, Liu et al., 2016, Fang et al., 2016, Rong et al., 2016).

Auricular tVNS has also shown promising analgesic effects. Examination of the effects of auricular tVNS on various somatosensory modalities in healthy participants shows that, compared to sham, tVNS significantly decreased pain intensity ratings in response to tonic heat pain, decreased mechanical pain sensitivity, and increased mechanical and pressure pain thresholds (Busch et al., 2013). Warm, cold, and mechanical detection thresholds were unaffected, indicating that tVNS specifically modulates pain processes of somatosensation. Frøkjær et al. (2016) observed a significant increase in bone pain (tibia pressure) thresholds in healthy participants, but no effects were observed in muscle pain thresholds and conditioned pain modulation. In a pilot study on chronic pelvic pain patients, a significant reduction of evoked pain intensity and temporal summation of mechanical pain compared to baseline, and a trending reduction compared to control stimulation, was observed after one session of respiratory-gated tVNS. No effects on clinical pain or the affective component of pain (i.e., unpleasantness) were observed, but it is likely that multiple sessions of tVNS are required in order to ascertain meaningful results, particularly in chronic pain patients. Nevertheless, affect was modulated by tVNS as patients experienced a significant anxiolytic effect (Napadow et al., 2012).

Using tVNS stimulation parameters similar to those used during electroacupuncture (see Table 2), Laqua et al. (2014) did not observe an overall effect on pain threshold (electrical stimulation to the finger) with tVNS compared to placebo. Compared to baseline, a significant increase in pain threshold was observed in fifteen participants, while six participants responded with a significant decrease in pain threshold. These effects were not observed with the placebo condition. Based on these findings, the authors concluded that tVNS produced an anti- and pro-nociceptive response, respectively. The variable response could be attributed to either, or a combination of, the fluctuating stimulation parameters, the intensity of the stimulation (although not reported), or the area of the ear being stimulated, i.e., the cavum conchae, which is innervated by not only the ABVN but also, and more so, by the greater auricular nerve (Peuker and Filler, 2002). It is also plausible that the mood state of the participants influenced their pain perception; however, mood was not reported in this study. The same group recently reported similar findings in an fMRI study using a similar paradigm but different stimulation parameters. Overall, no significant effects on heat pain thresholds were observed pre- vs. post-tVNS or placebo. However, subgroups with a significant increase or decrease in pain threshold in response to tVNS were once more found (Usichenko et al., 2016). The variable responses could, again, be due to the stimulation parameters, the region of ear stimulation, or the participants’ mood state. Nevertheless, the consistent diametrically opposite responses to vagus stimulation that occurred in both studies, and lack of non-responders, supports the notion that the vagus nerve alters pain perception.

**Table 2:** Summary of transcutaneous vagus nerve stimulation (tVNS) studies.

Author	Sample	Parameters	Main outcomes	
			Pain	Affect
Barbanti et al. (2015)	Migraine n = 50	At attack onset: two 120 s doses of electrical stimulation (parameters not reported), 3 min interval, over 2 weeks	↑ pain relief	Not reported
Busch et al. (2013)	Healthy n = 48	25 Hz, 0.25 ms pw, 1.6 mA ± 1.5 mA 1 h continuous	↓ tonic heat pain intensity; mechanical/pressure pain thresholds, mechanical pain sensitivity ns wind-up; thermal detection/pain thresholds; mechanical detection threshold	Not reported
Frøkjær et al. (2016)	Healthy n = 18	30 Hz, 250 μs pw 1.46 mA ± 0.73 mA (final) 1hr	↑ bone pain threshold ns muscle pain threshold	Not reported
Goadsby et al. (2014)	Migraine n = 30	At moderate to severe pain: two 90 s doses of electrical stimulation (parameters not reported), 15 min interval, over 6 weeks	↓ migraine pain	Not reported
Kinfe et al. (2015)	Migraine n = 20	1 ms burst 5-kHz pulse at 25 Hz, 0 to 24 V for 2 min = 1 dose. Prophylactic: 4 doses/day Acute: 2 doses	↓ headache intensity/frequency ↓ migraine attacks ↑ pain relief	↓ depression
*Komisaruk et al. (1997)	Complete spinal cord injury n = 16 Control n = 5	12 min vaginocervical stimulation	↑ pain detection threshold ↑ pain tolerance threshold	Not reported
Laqua et al. (2014)	Healthy n = 22	2–100 Hz bursts, 0.2 ms pw 30 min	ns overall ↑↓ pain thresholds	Not reported
Napadow et al. (2012)	Chronic pelvic pain n = 15	30 Hz, 450 μs pw 0.5 s respiratory-gated stimulus 0.43 ± 0.25 mA	↓ pain intensity, temporal summation ns clinical pain	↓ anxiety ns pain unpleasantness
Nesbitt et al. (2015)	Cluster headache n = 19	Five 5-kHz pulses at 25 Hz for 2 min = 1 dose. Prophylactic: up to 3 doses Up to 3 doses/attack	↓ headache frequency	Not reported
*Sedan et al. (2005)	Healthy n = 31	Ingested 1500 ml of water within 10–12 min	↑ heat pain threshold ↓ tonic heat pain, laser pain ns mechanical pain threshold, temporal summation	Not reported
Straube et al. (2015)	Migraine n = 46	25 Hz or 1 Hz (control), 250 μs pw 30 s on/30 s off 4 h/day, 3 mos	↓ headache frequency (1 Hz > 25 Hz) ns headache intensity	Not reported
Usichenko et al. (2016)	Healthy n = 20	8 Hz, 200 μs pw mean: 7.6 mA (range 5.0–11.5)	ns overall ↓↑ heat thresholds	Not reported

↓, decrease.

↑, increase.

ns, non-significant.

pw, pulse width.

* Stimulation of the vagus nerve was performed non-electrically.

Interestingly, a recent 3-month randomized controlled clinical trial investigating the treatment of chronic migraine with high (25 Hz) vs. low (1 Hz) frequency tVNS found an overall reduction in headache days per 28 days but found a significantly larger reduction with *low* frequency tVNS, which was the control stimulus (Straube et al., 2015). The greater response to the low frequency stimulation seemingly contradicts previous human and animal studies indicating that low frequency vagal stimulation facilitates pain. However, as the authors point out, analgesic effects in response to low frequency stimulation of other nerves (e.g., spinal or trigeminal) have been shown to induce long-term depression in the spinal system and craniofacial area (Ellrich and Schorr, 2004, Yekta et al., 2006, Aymanns et al., 2009). In the latter case, the resulting suppression of activity in the spinal trigeminal nucleus (STN), a projection of the NTS, could reduce migraine pain. Functional brainstem imaging data shows that auricular tVNS at an equivalent high frequency (25 Hz) activated the STN (Frangos et al., 2015), while non-invasive VNS via the neck (described below), significantly reduced STN activity (Frangos and Komisaruk, 2017) using parameters that are effective against migraine but similar to auricular tVNS.

This more recent approach of tVNS that has also been shown to access central vagal projections (Ay et al., 2015, Frangos and Komisaruk, 2017) works via mild electrical stimulation of the external surface of the neck over the region of the cervical vagus nerve. Using this approach, multiple studies show significant improvement in cluster headache and migraine when used prophylactically and acutely. Reductions in attack frequency, headache days, and depression have been observed. In some cases, nearly half of the attacks were aborted approximately 10 min after tVNS (Goadsby et al., 2014, Barbanti et al., 2015, Kinfe et al., 2015, Nesbitt et al., 2015).

In a non-invasive, physiological approach of stimulating the vagus nerve, Sedan et al. (2005) reported that gastric distention, induced by rapidly drinking 1500 ml of water to activate vagal afferents, significantly increased heat pain thresholds and decreased sensitivity to tonic heat pain induced by laser. However, mechanical pain threshold and sensitivity to mechanical temporal summation were not significantly affected. Vaginocervical stimulation has also been shown to produce significant analgesic effects that are hypothesized to be mediated by the vagus nerve. These effects were observed in complete spinal cord injured women. The vagus nerve would provide the necessary afferent pathway, as it bypasses the spinal cord and projects directly to the brain (Komisaruk et al., 1997). A subsequent brain imaging study in spinal cord injured women provided evidence in support of this pathway, as vaginal stimulation activated the NTS (Komisaruk et al., 2004).

Overall, tVNS is showing promising results against experimental and chronic pain. Ten of the 12 tVNS studies discussed in this section and summarized in Table 2, reported reduced pain, while the other two studies had subgroups experiencing both increased and decreased pain perception. The field is in its infancy and well-controlled, longitudinal studies using comparable parameters to investigate the effects of VNS across all sensory modalities are still required. In order to better characterize the effects of VNS on pain, psychological measures must be included in future studies. Table 1, Table 2 convey the dearth of information available about the impact VNS has on the affective component of pain, despite the evidence that affect is modulated by VNS (Cimpianu et al., 2017), and that affect modulates pain (Bushnell et al., 2013). Reducing the affective component of a painful sensation by improving mood, for example, may be another mechanism by which vagus stimulation attenuates pain. The psychological factors that modulate pain are discussed below.

## The effects of psychological factors on pain perception

Robust evidence indicates that psychological factors such as mood, emotions, and attention modify pain perception (for review, refer to Bushnell et al., 2013). Successful experimental manipulations of mood through, e.g., films, music, images, and odors, have been shown to significantly affect pain perception such that positive mood inducing stimuli attenuate pain, while negative stimuli increase pain (Cogan et al., 1987, Zelman et al., 1991, Weisenberg et al., 1998, Meagher et al., 2001, Villemure et al., 2003). Anxiety-inducing stimuli have been shown to decrease pain threshold and induce hyperalgesia (Rhudy and Meagher, 2000, Ploghaus et al., 2001). Chronic pain patients are also detrimentally affected by negative emotional states and attitudes that fluctuate on a daily basis and exacerbate their pain symptoms (Haythornthwaite and Benrud-Larson, 2000, Schanberg et al., 2000).

Distinct psychological factors can modulate pain perception differentially. Pain perception can be divided into a sensory component that can be measured by the intensity of the stinging, burning, and aching sensations, and an affective component that can be measured by the unpleasantness of those sensations. Attentional modulation of pain has distinct effects on pain perception compared to mood. The role of attention in pain modulation preferentially affects perceived pain intensity, whereas mood, or affective state, preferentially modulates the unpleasantness of pain (Villemure et al., 2003, Loggia et al., 2008). Villemure and Bushnell (2009) elucidated the dissociable neural networks of attention and mood underlying the modulation of pain intensity and unpleasantness, respectively. Focusing on odorants during pain stimulation decreased pain intensity ratings along with the pain-induced activity in the anterior insula. The decreased pain intensity ratings correlated with entorhinal cortex and superior posterior parietal cortex activity, suggesting they may be key attention-related pain modulatory regions. The positive mood-inducing odors (independent of attention) decreased unpleasantness and pain-related activation within the anterior cingulate cortex (ACC), thalamus, and primary (S1) and secondary (S2) somatosensory cortices. The decreased unpleasantness ratings correlated with the activity in the lateral inferior frontal cortex and the periaqueductal gray (PAG) area, which suggests they are emotion-related pain modulatory regions (Villemure and Bushnell, 2009).

## The effects of VNS on psychological factors

### VNS and affect

The psychological effects of iVNS, particularly on depression, have been well examined, as iVNS is currently an FDA-approved therapy against treatment-resistant depression (Rush et al., 2000, Sackeim et al., 2007). Positive effects on other conditions such as panic disorder, obsessive-compulsive disorder, and post-traumatic stress disorder in response to iVNS have also been observed (George et al., 2008). Cimpianu et al. (2017) provide a systematic review of the effects of invasive and non-invasive VNS on psychiatric conditions. The review is predominantly composed of iVNS studies, and therefore, we will focus mainly on tVNS and affect.

Significant beneficial effects against depression, anxiety, and overall mood and well-being have been observed in healthy and chronic pain patients using auricular tVNS (Kraus et al., 2007, Hein et al., 2013, Napadow et al., 2012) and tVNS via the neck (Kinfe et al., 2015). Functional brain imaging studies have reported tVNS induced changes in brainstem and limbic regions that may account for the observed positive effects (Kraus et al., 2007, Kraus et al., 2013, Dietrich et al., 2008, Frangos et al., 2015, Frangos and Komisaruk, 2017, Yakunina et al., 2016). Of particular interest is the tVNS-induced activation of the locus coeruleus and raphe nuclei, which release norepinephrine and serotonin, respectively, and have significant modulatory roles in pain perception and psychological states and processes.

Auricular tVNS has recently been shown to significantly reduce depression and anxiety in patients with major depressive disorder (MDD) after one month of use compared to sham. Reduction of the clinical symptoms significantly correlated with an increase in resting-state functional connectivity between the right amygdala and left dorsolateral prefrontal cortex, two regions that have been previously implicated in MDD (Liu et al., 2016, Rong et al., 2016). Functional connectivity between the default mode network (DMN) and brain regions associated with emotion regulation (i.e., the insula and parahippocampus) have also reportedly decreased after one month of tVNS compared to sham stimulation in MDD patients. The change in depression severity significantly correlated with functional connectivity changes between the DMN and regions that are implicated in both pain modulation and emotion such as the anterior insula and ACC (Fang et al., 2016).

The behavioral and brain imaging studies above provide supporting evidence that tVNS improves affect and produces functional changes in brain regions where pain modulation and affect converge. Thus, it is possible that investigations of vagal pain modulation may not be capturing the preferential modulation of affect on pain unpleasantness, as these factors are not reliably reported on (Table 1, Table 2).

### VNS and cognition

Evidence of vagal effects on cognition are discussed below and lend support towards the inclusion of attentional measures (which preferentially modulate pain intensity) when investigating vagal pain modulation.

Given the invasive nature of iVNS, a limited number of studies exist solely on the effects of iVNS on cognitive functions. Improvements in motor speed, psychomotor function, verbal fluency, and executive functions such as logic reasoning and working memory have been observed in patients with depression receiving iVNS (Sackeim et al., 2001). Enhanced recognition memory in a verbal task was observed in epileptic patients with iVNS and a similar enhanced retention performance on an inhibitory-avoidance task had been previously observed in rats (Clark et al., 1995, Clark et al., 1999). Increased daytime alertness and vigilance has also been reported in epileptic patients with iVNS and seems to be frequency dependent such that low frequency iVNS produces the increased attentional effect, while high frequency stimulation induces somnolence (Malow et al., 2001, Galli et al., 2003, Rizzo et al., 2003, Serdaroglu et al., 2016).

The extent to which tVNS modulates cognitive functions aside from depression and anxiety is still emergent. In a study on healthy older individuals, acute tVNS compared to sham enhanced associative memory performances on a face-name task (Jacobs et al., 2015). tVNS has also been shown to accelerate fear extinction learning, however a return of fear was observed 24 h later (Burger et al., 2016). Fear extinction effects in response to vagus stimulation had been previously observed in rats (Peña et al., 2014). In studies using tVNS to modulate the GABA and norepinephrine pathways, tVNS has been shown to increase reaction time and response selection during action cascading (Steenbergen et al., 2015) and to modulate inhibitory control processes during high working memory tasks compared to low working memory tasks (Beste et al., 2016). By contrast, Sellaro et al. (2015) found no effect of tVNS on a reaction time task. However, post-error slowing, which is partially mediated by the noradrenergic system, significantly increased with tVNS compared to sham.

Based on previous studies showing that cognitive processes such as attention preferentially modulate pain intensity such that increased attention to pain increases pain perception, it is possible that some of the pain facilitatory effects of VNS are a result of increased attention to the pain stimulus, a sensation which is inherently attention demanding (Miron et al., 1989). In addition, tVNS produces increased activity in brain regions associated with interoceptive processes such as the insula (Critchley et al., 2004, Kraus et al., 2007, Kraus et al., 2013, Dietrich et al., 2008, Frangos et al., 2015, Frangos and Komisaruk, 2017), which may result in greater directed attention towards bodily sensations during the *on* periods of VNS, compared to the *off*, as seen in the findings of Borckardt et al. (2006). However, it is also important to point out that the analgesic effects reported by Kirchner et al. (2000) were independent of the on/off cycle of the vagal stimulator.

The trending positive effects of VNS on various cognitive processes, including attention, are further indication that psychological factors should be considered in studies investigating vagal pain modulation.

## Mechanisms of vagal modulation of pain

Here, we present a summary of the animal literature and human brain imaging studies that elucidate portions of the mechanism of action of vagus nerve stimulation.

The pro- and anti-nociceptive effects of VNS were elucidated in early animal studies. A comprehensive review by Randich and Gebhart (1992) describes the complexity of cervical, thoracic, and cardiac vagal modulation of nociception and the differential effects that electrical stimulation parameters have on inhibition and facilitation of nociception. In the tail-flick reflex test on rats, low intensity stimulation (between 2.5 and 20 μA) produced a facilitatory effect, while high intensity stimulation (≥30 μA) produced inhibition of the reflex. Inhibition, but not facilitation, was found to be dependent on intensity, frequency (no less than 20 Hz), and pulse width (no less than 2 ms) of the stimulation (Ren et al., 1988, Ren et al., 1991). Unfortunately, optimal stimulation parameters observed in animal studies are not directly translatable to humans, which may account for the lack of consistency of stimulation parameters across human studies.

The anti-nociceptive effects of VNS seem to be primarily dependent on the NTS and its projections to the locus coeruleus and raphe nuclei, followed by the subsequent activation of the descending noradrenergic and serotonergic systems in the spinal cord, including spinal opioid receptors, all of which inhibit second order nociceptive neurons in the spinal cord (Basbaum and Fields, 1978, Randich and Aicher, 1988, Ren et al., 1990). Nishikawa et al. (1999) later reported VNS-induced activation of an ascending pain inhibitory pathway from the PAG and raphe nuclei to the ventral posteromedial nucleus of the thalamus. In addition to norepinephrine, serotonin, and opioids, GABA has also been implicated as a possible mediator of VNS-induced analgesia, as it was present in the cerebral spinal fluid of epilepsy patients receiving iVNS (Ben-Menachem et al., 1995) and has been implicated in pain reduction with transcutaneous electrical nerve stimulation (TENS) treatments (Maeda et al., 2007, Johnson and Bjordal, 2011). Trigeminal nociception has been counteracted by both invasive and non-invasive VNS as measured by a reduction of formalin-induced Fos-expression in the STN with a reduction of pain-related behavior on the side of the facial nociceptive stimulus (Bohotin et al., 2003), and a reduction of STN extracellular glutamate induced by glyceryl trinitrate, a headache trigger (Oshinsky et al., 2014). Vagal stimulation may also reduce nociception by activating propriospinal neurons in cervical spinal segments 1–3 that project to, and inhibit, spinothalamic tract neurons below C3 (Zhang et al., 1996, Zhang et al., 2003, Chandler et al., 2002). Access to propriospinal neurons may be possible via a small percentage of vagal afferent fibers from the nodose ganglion that project to the upper cervical spinal cord (McNeill et al., 1991).

Evidence of vagal access to the descending and ascending pain inhibitory pathways elucidated in animal studies is also supported by human tVNS functional MRI studies that report activation of the NTS, raphe nuclei, locus coeruleus, and periaqueductal gray, as well as other regions implicated in pain modulation such as the nucleus cuneiformis and dorsolateral prefrontal cortex (Dietrich et al., 2008, Frangos et al., 2015, Yakunina et al., 2016, Frangos and Komisaruk, 2017). Imaging studies on iVNS and tVNS report activity within other vagal projection sites such as the insula, thalamus, amygdala, hippocampus, postcentral gyrus, nucleus accumbens, hypothalamus, and prefrontal cortex (Bohning et al., 2001, Ring et al., 2000, Van Laere et al., 2000, Vonck et al., 2000, Lomarev et al., 2002, Narayanan et al., 2002, Liu et al., 2003, Nahas et al., 2007, Dietrich et al., 2008, Kraus et al., 2007, Kraus et al., 2013, Frangos et al., 2015, Frangos and Komisaruk, 2017). Many of these regions also respond to and modulate pain, e.g., the insula, somatosensory cortices, thalamus, and prefrontal cortex (Apkarian et al., 2005, Bushnell et al., 2013). Recently, pain responsive brain regions were reportedly modulated by tVNS (Usichenko et al., 2016). In response to painful thermal stimulation, the insula, thalamus, and ACC were activated, and when coupled with tVNS, the activity in the ACC decreased, while amygdala activity increased. Activity in the secondary somatosensory cortex (S2), posterior insula, ACC, and caudate nucleus was correlated with the heat stimulation, while only right anterior insula activity correlated with the heat stimulation during tVNS. The anterior insula has been implicated in modulating the perception of pain. Specifically, shifting attention away from pain decreases pain intensity and pain-evoked activity in the anterior insular cortex (Villemure and Bushnell, 2009).

Inflammatory conditions that produce or are induced by pain may also improve with VNS as descending vagal signals activate anti-inflammatory pathways that suppress secretion of pro-inflammatory cytokines (e.g., TNFα and IL-1β, Borovikova et al., 2000), which could subsequently ameliorate associated pain. Yuan and Silberstein (2016) provide an up-to-date review on VNS and the anti-inflammatory response.

## Proposed model of vagal pain modulation

**Fig. 2:**
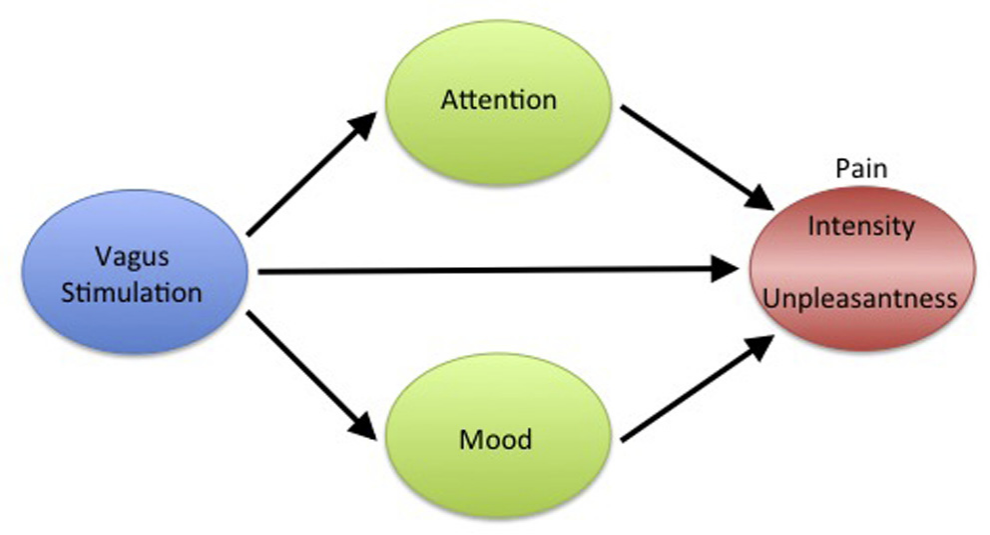
Model of vagal pain modulation. Stimulation of the vagus nerve can modulate pain directly through the descending pain inhibitory system, through attentional modulation that can preferentially modulate pain intensity, and/or through induced mood changes that can preferentially modulate the unpleasantness associated with pain.

The studies summarized in Table 1, Table 2 provide evidence that stimulation of vagal afferents produces significant, and in some cases, clinically significant analgesic effects in healthy participants and patient populations. Nevertheless, some discrepancies exist and further work is necessary in order to fully elucidate the mechanism by which VNS modulates pain perception in both favorable and unfavorable directions. To this end, we propose a new model that includes psychological variables that have previously been shown to modulate pain perception and that are also modulated by vagal stimulation. With few exceptions, investigators have focused on the effects of VNS on psychological factors *or* on pain. Future studies investigating vagal modulation of pain should include measures of psychological factors, such as mood and attention, as they significantly interact with the way pain is perceived. Fig. 2 depicts the direct effects that VNS has on pain perception, as well as the psychological variables (i.e., attention and mood) that can differentially modulate the components of pain (i.e., intensity and unpleasantness). Inclusion of these factors may better characterize the role of the vagus nerve in the perception of pain, and may help resolve the discrepancies in the literature.

Additional factors to consider that may also result in discrepant findings are the duration, parameters, and location of VNS stimulation. It is worth noting that many of the VNS studies described above are testing acute effects of vagal stimulation, which may produce immediate significant changes in pain responsive brain regions but not necessarily behavioral responses, as observed in the study by Usichenko et al. (2016). Four-weeks of tVNS reduced clinical symptoms in MDD patients and produced resting-state functional connectivity changes in associated neural-networks (Fang et al., 2016). Thus, more longitudinal studies are required to gain a better understanding of the potential persisting affects of vagal stimulation.

While increased pain perception in response to VNS may be a function of stimulation parameters, it is also likely that possible increased attention, alertness, and vigilance in response to VNS may increase attention to evoked pain and thereby increase pain intensity. An occurrence of increased pain perception in response to VNS is, indeed, a response and evidence of vagal modulation of pain. Thus, participants with such reactions should not be deemed “unresponsive”, as that would, by definition, indicate that VNS neither increased nor decreased pain perception. Pro-nociceptive effects in response to vagal stimulation are intriguing phenomena that warrant further examination.

It is also possible that in a true case of unresponsiveness, as measured by pain threshold or pain intensity ratings, VNS may, indeed, be having an effect on a component of pain that is not being measured, i.e., the unpleasantness associated with pain, which can be independent of the perceived pain intensity. In other words, it is possible that in cases where VNS either increases or has no effect on pain (as measured by intensity), it may also be decreasing the unpleasantness associated with the pain, as noted in the anecdotal comments of one of the patients in a previous study (Borckardt et al., 2005) who was no longer “bothered” by his existing pain. Furthermore, a decrease in pain unpleasantness in response to VNS may be attributed to improved affect, which evidently has not been reliably measured in studies investigating the effects of VNS on pain. To this effect, based on the findings reported in Usichenko et al. (2016), one could argue that tVNS may have possibly increased mood and decreased pain unpleasantness as the activity in the secondary somatosensory cortex, which is associated with coding the affective component of pain, i.e., unpleasantness, was no longer associated with the painful heat stimulation during tVNS.

In conclusion, the present studies discussed provide preliminary evidence in humans that stimulation of the vagus nerve alters the way pain is perceived and the findings corroborate early reports in animals. The present studies also provide evidence that the vagus nerve alters psychological processes that are known to modulate pain perception differentially. It remains unclear as to whether VNS-induced psychological effects partially mediate vagal pain modulation. However, evidence of vagal-induced analgesia and vagal-induced improvement in mood, together with the known psychological effects on pain perception strongly indicate a need for including psychological measures in future studies investigating the effects of vagus nerve stimulation on pain.

## Funding

This research was supported by the Intramural Research program of the NIH, National Center for Complementary and Integrative Health.

## Conflict of interest

There is no conflict of interest for any of the authors.
